# 3D Computational Modeling of Fe_3_O_4_@Au Nanoparticles in Hyperthermia Treatment of Skin Cancer

**DOI:** 10.2147/NSA.S495377

**Published:** 2025-04-12

**Authors:** Piotr Gas, Muhammad Suleman, Farah Khaliq

**Affiliations:** 1 Department of Electrical and Power Engineering, Faculty of Electrical Engineering, Automatics, Computer Science and Biomedical Engineering, AGH University of Krakow, Krakow, Poland; 2 Department of Mathematics, Riphah Institute of Computing & Applied Sciences (RICAS), Riphah International University, Lahore, Punjab, Pakistan

**Keywords:** skin cancer, gold-iron oxide magnetic nanoparticles, magnetic hyperthermia, thermal therapy, mathematical modeling, tumor damage, finite element method (FEM)

## Abstract

**Background:**

Nanotechnology can be used to treat a diversity of cancers with different physiological properties. Skin cancers are common among people affected by an excessive solar radiation of the ultraviolet (UV) range.

**Introduction:**

This paper describes a mathematical formulation and simulation approach for the magnetic hyperthermia therapy of skin cancer using gold-coated iron oxide (Fe_3_O_4_@Au) magnetic nanoparticles (MNPs).

**Methods:**

The authors created an artificial 3D geometry model of skin cancer with tissue-mimicking materials, constructed a mesh, and solved all the required physics for electro-thermal simulation using FEM-based software. The heat transfer in the skin tissue was modeled using the Pennes bioheat equation, and the Helmholtz-type equation of quasi-static magnetic field produced by a three-turned coil surrounding the tumor.

**Results:**

The simulated magnetic field pattern was compared with that of the analytical solution along the symmetry axis of the helical coil with good agreement. The obtained results show that the tumor damage is maximum in the tumor center and decreases towards its outer boundaries. Additionally, the impact of varying values of blood perfusion rate, blood density, blood specific heat capacity, heat dissipation produced by Fe_3_O_4_@Au MNPs, and metabolic heat generation has been examined for thermal therapy. The performed simulations show that all these parameters influences heating characteristics of tumor tissues by gold-coated magnetic nanoparticles.

**Conclusion:**

Gold-iron oxide magnetic nanoparticles succeeded to damage 90–99% skin cancer. Among all the contributing parameters, the blood perfusion is the most sensitive parameter in thermal therapy of skin tumor.

**Recommendations:**

On the bases of results obtained, we recommend physicians to use Fe_3_O_4_@Au MNPs in real time medical skin cancer treatments.

## Introduction

Cancer treatment remains a difficult problem that has inspired many scholars worldwide, despite advancements made over the past two decades. New therapeutic options are constantly being developed, and they have the potential to increase patient quality of life, survival rates, and therapeutic effectiveness, while reducing negative side effects.^1^ Skin cancer accounts for approximately 30% of all newly diagnosed malignancies worldwide. In 2022 sees over 1.5 million new cases of skin carcinomas, including 331,722 cases of melanomas and other non-melanoma skin cancers (NMSCs).^2^ Most new cases of melanoma were diagnosed in Europe (44.1%) and NMSCs in North America (49.2%). Most deaths due to melanomas occur in Europe (44.6%) and NMSCs in Asia (46.1%). Every year, three million people in the United States are affected by skin cancer.^3^ It is projected that by 2025, approximately 6.7% of new cases of skin cancer will the word compared to 2023.^2^ Among these, 353,947 were melanomas, and 62,551 died from this disease.

The largest organ in the human body is the skin, which accounts for 8% of the total body mass of an adult and has a surface area of approximately 1.8 m^2^.^4^ Owing to its powerful barrier abilities, the skin maintains body temperature, prevents salt and fluid loss, and limits evaporation.^5^ Specifically, reactive oxygen species (ROS) that cause photo ageing are produced when the skin is continuously exposed to numerous threats from the environment, including ultraviolet (UV) radiation, chemicals, and pathogenic microorganisms,^6^ which are mostly caused by prolonged exposure to direct sunlight.^7^ Patients with skin cancer exhibit normal indications of enduring sun damage, such as collagenases, inconsistent pigmentation, wrinkles, telangiectasia, and solar keratosis in sun-exposed areas.^1^ The three most prevalent types of skin cancers are melanoma (MSC), squamous cell carcinoma (SCC), and basal cell carcinoma (BCC).^8^ MSC cause the highest percentage of deaths, whereas NMSCs are the most commonly diagnosed type of cancer.^1^ BCC and SCC are non-malignant tumors that cause relatively little metastasis, whereas MCS has a significant possibility of metastasizing to other parts of human body.^9^,^10^

Hyperthermia therapy is a technique in which the tumor temperature is raised to the therapeutic range of about 44–45°C to damage cancerous cells.^11–19^ Gold possesses various characteristics such as adjustable sizes, simple production, simple modification, surface plasmon resonance (SPR), and diverse morphology, which includes stars, hexapods, spheres, rods, etc.^20^,^21^ Due to these properties, gold nanoparticles (GNPs) as theragnostics are very attractive in numerous biomedical applications including nanoparticle hyperthermia, resonance imaging, tumor drug delivery and tumor ablation.^22–24^ The gold coated magnetic nanoparticles (GMNPs) are permitted to concentrate on the skin tumors and then excited by an external alternating (AC) magnetic field to generate thermal effect in the treated region.^25–27^ GMNPs consume electromagnetic (EM) power and transform it into heat, which increases tumor temperature and results in malignant cell damage.^28^,^29^ GNPs can be manufactured in a variety of sizes and used for optimal tumor penetration and distribution.^30–35^ Moreover, GNPs are good radiosensitizers that may be used in brachytherapy combined with radiation therapy to enhance the effectiveness of melanoma skin cancer (MSC) treatment.^36^,^37^ Other melanoma treatments are describe in similar articles.^38–40^

The number of prominent researchers, who became famous for their research on gold magnetic nanoparticles (GNPs) for skin cancer treatment, is highlighted below. Bagheri et al^41^ studied the application o GNPs in the treatment of cancer, both in vivo and in vitro. They found that these nanoparticles have a huge potential for treating MSC. Jia et al^22^ studied the in vivo and in vitro toxicity of GNPs in biological systems, focusing mainly on their unique physicochemical properties, such as particle size, shape, surface charge, and their modifications. Moreover, they analyzed the effects of GNPs on both metabolism and the immune system. Kanavi et al^37^ described an in vitro study of choroid melanoma and Burkitt’s lymphoma cells treated with GNPs during continuous gamma irradiation for ocular cancer therapy. Lee et al^42^ examined the toxicity and side effects of GNPs for breast cancer treatment. They pointed out that non-magnetic GNPs in the form of nanoshells and nanorods can induce hyperthermic effects, but only under near-infrared (NIR) wavelengths of electromagnetic radiation, typical for laser irradiation during photothermal therapy, owing to the SPR effect of such NPs.^43^ Moreover, NIR laser-induced hyperthermia causes localized heating of tumor-injected GNPs and reduces the risk of overheating the surrounding normal tissues.^44^ Most synthetic GNP coating agents induce strong inflammatory pathways, thus limiting their use during in vivo photothermal therapy. De Matteis et al^45^ produced stable polyphenol-capped AuNPs with enhanced anti-inflammatory and anticancer properties. Lim et al^21^ reviewed current methods for the diagnosis, monitoring, and treatment of animal cancers with GNPs. They pointed out that these particles could act as potential drug delivery agents and nanoheaters in plasmonic photothermal therapy for tumor eradication. Visaria et al^46^ developed a new nanoparticle delivery system including 33-nm polyethylene glycol-coated (PT-cAu-TNF-A) GNPs with the incorporated cytokine TNF-α to enhance tumor damage. They noticed that GNPs injected into mouse mammary carcinomas during combined hyperthermia therapy significantly reduced tumor growth, cancer cell survival, and tumor blood perfusion at TNF-α doses that were found to be toxic with GNPs treatment alone. Hainfeld et al^36^ confirmed the effectiveness of GNPs as excellent absorbers of X-rays and enhancers in radiotherapy. During animal trials they injected into mouse mammary tumors before irradiation, and the effects of radiotherapy combined with GNPs were observed. They noticed that approximately 86% of the subcutaneous tumors were cured long-term with conjugated therapy, whereas only 20% were cured with radiation alone.

New therapeutic possibilities for the treatment of skin cancer are provided by the use of gold-coated magnetic nanoparticles (GMNPs).^47^ Pandesh et al^48^ synthesized Fe_3_O_4_@Au core-shell MNPs and used them in magnetically targeted nano-photothermal therapy for mouse melanoma. The tests were performed for the following groups: (1) control group, (2) GMNPs alone, (3) laser alone, (4) GMNPs plus laser, and (5) GMNPs plus 3500 Gauss magnet plus 808 nm wave diode laser. After two weeks of treatment, the highest inhibition rates (IRs) were recorded for tumors treated with the MNP-magnet-laser. In this group, the mean tumor volume increased from 99 mm^3^ to 768 mm^3^, which resulted in the lowest increase (7.7 times) in all tested groups compared to the control group. Nori et al^49^ prepared gold-coated magnetite MNPs (Fe_3-δ_O_4_@Au) decorated with a thiol-containing dendrimer and tested them for treating human breast cancer (MCF-7) cell line with satisfactory effectiveness. Amin et al^50^ showed that Fe_3_O_4_@Au MNPs are moderately toxic (more toxic than normal Fe_3_O_4_ MNPs) but can be safely used in the treatment or diagnosis of skin cancer, which was confirmed by tests on human squamous carcinoma (A431) cells. Kazem et al^51^ conducted research on mice using gold-coated iron oxide nanoparticles (Au@IONPs) divided into five groups: (1) normal mouse, (2) mouse with MSC, (3) mouse with MSC treated by GMNPs (0.1 mL nanoparticle suspension with an Au concentration of 50 μg/mL and Fe concentration of 100 μg/mL, 0.4 T magnet per 2 h for tumor targeting) alone, (4) mouse with MSC treated by EBRT (dose 8 Gy, energy 6 MeV), and (5) mice with MSC treated with EBRT plus GMNPs. The authors discovered that Au@IONPs with magnetic targeting before EBRT reduced tumor growth the most after 21 days of treatment compared to the control group. Additionally, they showed that GMNPs were not toxic to mice and were deposited mainly in the tumor tissue. In conclusion, it can be said that the metallic (gold) element of nanostructure is responsible for heating the cancer tissue in photothermal therapy and enhances the chemo-sensitivity and radio-sensitivity of the tumor in combined therapy.^52–55^ In contrast, the iron part of the nanostructure was effective in tumor targeting under the magnetic field of the permanent magnet, and caused tumor heating under an external AC magnetic field, which is extremely important in targeted therapy and hyperthermia treatment of melanoma skin cancer.

**Figure 1:**
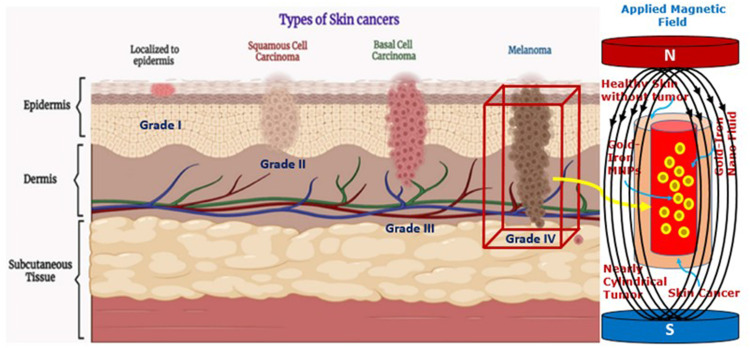
Strategy to treat skin cancer with gold iron-oxide magnetic nanoparticles.

**Notes**: Reproduced from Hasan N, Nadaf A, Imran M, et al. Skin cancer: understanding the journey of transformation from conventional to advanced treatment approaches, 595 Mol. Cancer. 2023;22:168. Copyright 2023, Springer Nature. Creative Commons Attribution 4.0 International License.^56^

Common treatment strategies for skin cancer include a surgical protocol, chemotherapy, or radiation therapy^56^ and each of these medical procedures carries the side effect of killing normal cells along with tumor cells. Moreover, the above-described literature shows that most of the authors worked on cancer treatments using in vivo, in vitro or analytical approaches. These reasons motivated us to conduct an in silico study. Contrary to costly and time-consuming in vivo and in vitro studies, we used computational techniques for skin cancer treatment planning available in computer-based simulations. Some of the aforementioned analytical studies deal with simplified geometries, and their analytical solutions fail to handle irregular shape geometries; therefore, we employed finite element method (FEM)-based software to solve the electro-thermal problem and to handle all complex geometries. An overview of this study is shown in Figure 1.

## Materials and Methods

### Materials

**Figure 2:**
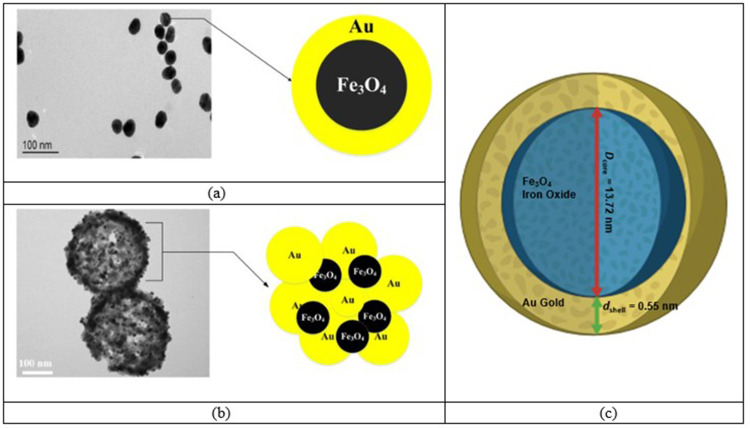
Gold coated iron oxide MNPs with magnetic core (Fe_3_O_4_) and gold shell (Au): Sample TEM micrographs of: (**a**) single Fe_3_O_4_@Au MNP; (**b**) Fe_3_O_4_@Au MNPs (reproduced from^57^); and (**c**) analyzed spherical core-shell model of Fe_3_O_4_@Au MNP. Reprodced from Ali Dheyab M, Abdul Aziz A, Jameel MS, Moradi Khaniabadi P. Recent advances in synthesis, medical applications and challenges for gold-coated iron oxide: comprehensive study. Nanomaterials. 2021;11:2147. © 2021 by the authors.Licensee MDPI, Basel, Switzerland. This article is an open access article distributed under the terms and conditions of the Creative Commons Attribution (CC BY) license.^57^

Researchers hae investigated the heating characteristics of gold-coated magnetic nanoparticles (Fe_3_O_4_@Au MNPs) over the past 15 years as well as the possibility of modifying the particle size, shape, and surface functions for a variety of material applications in biology, medicine, and science.^29^ In the 1970s and the 1980s, initial research on the biodistribution of colloidal gold revealed that, after being administered parentally, Fe_3_O_4_@Au MNPs were quickly absorbed by the liver, released through bile, and removed with feces, thus possessing good biocompatibility.^20^,^57^ Therefore, having good heating efficiencies and being more biocompatible, we are going to use these Fe_3_O_4_@Au MNPs in the current research for the treatment of skin cancer. Different properties of the Fe_3_O_4_@Au MNPs were obtained from the literature and are listed in Table 1. Later, these properties were used in Equation (9) to calculate the overall nanoparticle heat dissipation *Q*_nano_, generated by Fe_3_O_4_@Au MNPs. In this study, gold-coated iron-oxide MNPs were used to treat a skin cancer. Transmission electron microscopy (TEM) micrographs and visualization of the spherical core-shell model of the Fe_3_O_4_@Au MNPs are shown in Figure 2.

**Table 1:** Properties of Fe_3_O_4_@AuMNPs Used in the Simulation

Parameters	Description	Values	Units
*D*	Diameter of Fe_3_O_4_@Au MNP^58^,^59^	15	nm
*d*_shell_	Non-magnetic (gold) layer thickness of Fe_3_O_4_ MNP (see Equation (13) in Materials)	5.5	nm
*H*_0_	Magnetic field strength	1500	A/m
*f*	Frequency of the magnetic field^60^	571	kHz
*t*	Heating time	60	min
*M*_d_	Domain magnetization of magnetite*^61^	4.10 × 10^4^	A/m
*M*_s_	Saturation magnetization of magnetite*^61^	2.86 × 10^4^	A/m
μ_0_	Permeability of free space	4π × 10^–7^	H/m
k_B_	Boltzmann constant	1.38 × 10^–23^	J/K
*k*	Thermal conductivity of magnetite*^62^	40	W/m/K
*K*	Magnetic anisotropy constant for Fe_3_O_4_@Au MNPs^60^	2.0 × 10^4^	J/m^3^
*η*	Dynamic viscosity of magnetite*^61^	2.35 × 10^–3^	Pa∙s
ρ	Density of magnetite*^63^	5196	kg/m^3^
*ϕ*	Volume fraction of iron in magnetite*^61^	0.071	–
*C*_p_	Heat capacity of magnetite*^62^	670	J/kg/K

**Notes**: *for the magnetic core of Fe_3_O_4_@Au MNPs, the properties of magnetite were employed.

The ferrofluid considered in the current investigation consisted of both uncoated Fe_3_O_4_ and gold-coated Fe_3_O_4_@Au nanoparticles. Some important calculations regarding such a gold-iron oxide nanofluid are given below. Let *N*_Fe(U)_ be the number of iron atoms in the uncoated Fe_3_O_4_ nanoparticles, *N*_Fe(C)_ be the number of iron atoms in the gold-coated Fe_3_O_4_@Au nanoparticles, and *N*_Au_ be the number of gold atoms in the Fe_3_O_4_@Au nanoparticles. The total number of iron atoms in a ferrofluid composed of uncoated Fe_3_O_4_ and gold-coated Fe_3_O_4_@Au nanoparticles is given by:^63^

(1) $${N_{{\mathrm{Fe}}\left({\mathrm{T}} \right)}} = {N_{{\mathrm{Fe}}\left({\mathrm{C}} \right)}} + {N_{{\mathrm{Fe}}\left({\mathrm{U}} \right)}}$$

The ratios between iron and gold atoms in the Fe_3_O_4_@Au MNP ferrofluid were determined using direct current plasma–atomic emission spectroscopy (DCP-AES):^63^

(2) $${N_{{\mathrm{Fe}}\left({\mathrm{T}} \right)}} = {{56} \over {44}} {N_{{\mathrm{Au}}}}$$

(3) $${N_{{\mathrm{Fe}}\left({\mathrm{C}} \right)}} = {{47} \over {53}} {N_{{\mathrm{Au}}}}$$

Therefore, the fraction of uncoated Fe_3_O_4_ nanoparticles can be computed as follows:

(4) $${N_{{\mathrm{Fe}}\left({\mathrm{U}} \right)}} = {N_{{\mathrm{Fe}}\left({\mathrm{T}} \right)}} - {N_{{\mathrm{Fe}}\left({\mathrm{C}} \right)}} = \left({{{56} \over {44}} - {{47} \over {53}}} \right){N_{{\mathrm{Au}}}} = {{225} \over {583}}{N_{{\mathrm{Au}}}}$$

After dividing both sides of Equation (4) by *N*_Fe(T)_ we get:

(5) $${{{N_{{\mathrm{Fe}}\left({\mathrm{U}} \right)}}} \over {{N_{{\mathrm{Fe}}\left({\mathrm{T}} \right)}}}} = 1 - {{{N_{{\mathrm{Fe}}\left({\mathrm{C}} \right)}}} \over {{N_{{\mathrm{Fe}}\left({\mathrm{T}} \right)}}}} = 1 - {{517} \over {742}} = {{225} \over {742}} \approx 0.30$$

Thus the fraction of uncoated Fe_3_O_4_ nanoparticles in the ferrofluid is about 30.3%.

The average thickness of the gold shell (*d*_shell_) in the analyzed iron-oxide MNPs was estimated based on the metallic composition inside the spherical core-shell Fe_3_O_4_@Au MNP model presented in Figure 2c. For Fe_3_O_4_@Au MNPs, the atomic weight ratio of gold and iron atoms was estimated as:^63^

(6) $${\mathrm{AR}} = {{{N_{{\mathrm{Au}}}}} \over {3{N_{{\mathrm{Fe}}\left({\mathrm{C}} \right)}}}} = {1 \over 3} \cdot {{53} \over {47}} = {{53} \over {141}} \approx 0.3759$$

However, the atomic ratio of the gold and iron oxide particles can be computed as:

(7) $${\mathrm{AR}} = {{{N_{{\mathrm{Au}}}}} \over {3{N_{{\mathrm{Fe}}3{\mathrm{O}}4}}}} = {{{{{m_{{\mathrm{Au}}}}} \over {{M_{{\mathrm{Au}}}}}}} \over {3{{{m_{{\mathrm{Fe}}3{\mathrm{O}}4}}} \over {{M_{{\mathrm{Fe}}3{\mathrm{O}}4}}}}}}$$

where *m* and *M* are the mass and molar mass (molecular weight) of given material in the Fe_3_O_4_@Au MNP ferrofluid, respectively.

The total mass of iron oxide and gold in the ferrofluid can be calculated using the following formula:

(8) $${m_{{\mathrm{Fe}}3{\mathrm{O}}4}} = {V_{{\mathrm{Fe}}3{\mathrm{O}}4}}\,{\rho _{{\mathrm{Fe}}3{\mathrm{O}}4}} = {4 \over 3}{\mathrm{\pi }} R_{{\mathrm{core}}}^3\,{\rho _{{\mathrm{Fe}}3{\mathrm{O}}4}} = {{\mathrm{\pi }} \over 6}D_{{\mathrm{core}}}^3\,{\rho _{{\mathrm{Fe}}3{\mathrm{O}}4}}$$

(9) $${m_{{\mathrm{Au}}}} = {V_{{\mathrm{Au}}}}\,{\rho _{{\mathrm{Au}}}} = {{\mathrm{\pi }} \over 6}\left[{{{\left({{D_{{\mathrm{core}}}} + 2{d_{{\mathrm{shell}}}}} \right)}^3} - D_{{\mathrm{core}}}^3} \right] {\rho _{{\mathrm{Au}}}}$$

where *V* and ρ are the volume and mass density of the given material, *R*_core_ is the radius; *D*_core_ is the diameter of the magnetic core; and *d*_shell_ is the width of the gold shell of the core-shell Fe_3_O_4_@Au nanoparticles.

The molar masses of iron oxide nanoparticles and gold atoms are as follows: *M*_Fe3O4_ = 231.5326 g/mol and *M*_Au_ = 196.9666 g/mol.^64^

Fe_3_O_4_@Au MNPs of size 15 nm were used in this study. Let the diameter of magnetic core of Fe_3_O_4_@Au MNP is *D*_core_ = 13.90 nm (13.2 ± 0.7 nm^63^), density of iron-oxide core is ρ_Fe3O4_ = 5.196 g/cm^3^, and the density of gold shell is ρ_Au_ = 19.3 g/cm^3^.^63^ Therefore, the thickness of the gold shell, *d*_shell_ can be calculated using Equation (8), as follows:

(10) $${\mathrm{AR}} = {{\left[{{{\left({{D_{{\mathrm{core}}}} + 2{d_{{\mathrm{shell}}}}} \right)}^3} - D_{{\mathrm{core}}}^3} \right]{\rho _{{\mathrm{Au}}}}} \over {3D_{{\mathrm{core}}}^3\,{\rho _{{\mathrm{Fe}}3{\mathrm{O}}4}}}} \cdot {{{M_{{\mathrm{Fe}}3{\mathrm{O}}4}}} \over {{M_{{\mathrm{Au}}}}}}$$

which further implies:

(11) $${d_{{\mathrm{shell}}}} = {1 \over 2}\left[{\root 3 \of {1 + 3{\mathrm{AR}} \cdot {{ {\ }{\rho _{{\mathrm{Fe}}3{\mathrm{O}}4}} } \over {{\rho _{{\mathrm{Au}}}} }} \cdot {{ {M_{{\mathrm{Au}}}}} \over {{M_{{\mathrm{Fe}}3{\mathrm{O}}4}}}}} - 1} \right]{D_{{\mathrm{core}}}}$$

After substituting the above data and the AR value from Equation (7), we obtain

(12) $${d_{{\mathrm{shell}}}} = {1 \over 2}\left[{\root 3 \of {1 + 3\cdot{{53} \over {141}}\cdot{{5.196 } \over {19.3 }}\cdot{{196.9666} \over {231.5326}} - 1} } \right]\cdot13.90 \approx 0.55\ {\mathrm{nm}}$$

### Methods

Our methodology consists of firstly creating the modeling framework of the thermal therapy processes, secondly using the finite element method (FEM) based computational tool^65^ of Comsol Multiphysics software, thirdly simulating the coupled models, fourthly, analyzing and interpreting the obtained results and giving suitable recommendations based on this analysis. The detailed steps are given in the following subsections.

#### Magnetic Field Associated With Helical Coil

To analyze the magnetic field produced by the helical coil across the Fe_3_O_4_@Au MNPs embedded in the tumor, we begin with Ampere’s law as follows:^25^

(13) $$\nabla \times {\bf{H}} = {{\bf{J}}_{\mathrm{e}}} + {{\bf{J}}_{\mathrm{c}}} + {{\partial {\bf{D}}} \over {\partial t}}$$

where **H** is the vector of the magnetic field strength (A/m), **J**_e_ is the vector of the excitation current density (A/m^2^), **J**_c_ is the vector of the conduction current density (A/m^2^), and **D** is the vector of electric induction (C/m^2^). After substituting the material constants **J**_c_ = *σ***E, D** = ε_0_*ε*_r_ **E** and **B** = μ_0_*μ*_r_**H** we obtain:

(14) $$\nabla \times {1 \over {{{\mathrm{\mu }}_0}{\mu _{\mathrm{r}}}}}{\bf{B}} = {{\bf{J}}_{\mathrm{e}}} + \sigma {\bf{E}} + {{\mathrm{\varepsilon }}_0}{\varepsilon _{\mathrm{r}}}{{\partial {\bf{E}}} \over {\partial t}}$$

where *σ* is the electrical conductivity (S/m), µ_0_ = 4π×10^–7^ H/m is the permeability of free space, *µ*_r_ is the relative permeability, ɛ_0_ = 8.85×10^–12^ F/m is the permittivity of free space, and *ɛ*_r_ is the relative permittivity of a material. Furthermore, magnetic induction **B** (T) can be computed from the magnetic vector potential **A** (A/m) as:^18^

(15) $${\bf{B}} = \nabla \times {\bf{A}}$$

thus

(16) $$\nabla \times \left({{1 \over {{{\mathrm{\mu }}_0}{\mu _{\mathrm{r}}}}}\nabla \times {\bf{A}}} \right) = {{\bf{J}}_{\mathrm{e}}} + \sigma {\bf{E}} + {{\mathrm{\varepsilon }}_0}{\varepsilon _{\mathrm{r}}}{{\partial {\bf{E}}} \over {\partial t}}$$

On the other hand, from the Faraday’s law

(17) $$\nabla \times {\bf{E}} = - {{\partial {\bf{B}}} \over {\partial t}} = - {\partial \over {\partial t}}\left({\nabla \times {\bf{A}}} \right) = - \nabla \times {{\partial {\bf{A}}} \over {\partial t}}$$

which yields

(18) $$\nabla \times \left({{\bf{E}} + {{\partial {\bf{A}}} \over {\partial t}}} \right) = \nabla \times \left({ - \nabla \varphi } \right) = 0$$

where the quantity in brackets (gradient of electric potential) in most cases is equal to zero,^66^ thus:

(19) $${\bf{E}} = - {{\partial {\bf{A}}} \over {\partial t}}$$

Next, substituting Equation (19) into Equation (16) we obtain:

(20) $$\nabla \times \left({{1 \over {{{\mathrm{\mu }}_0}{\mu _{\mathrm{r}}}}}\nabla \times {\bf{A}}} \right) = {{\bf{J}}_{\mathrm{e}}} - \sigma {{\partial {\bf{A}}} \over {\partial t}} - {{\mathrm{\varepsilon }}_0}{\varepsilon _{\mathrm{r}}}{{\partial {\bf{A}}} \over {\partial t}}$$

For stationary cases, the derivatives are equal zero we finally get:^18^,^25^,^26^

(21) $$\nabla \times \left({{1 \over {{{\mathrm{\mu }}_0}{\mu _{\mathrm{r}}}}}\nabla \times {\bf{A}}} \right) = {{\bf{J}}_{\mathrm{e}}}$$

The vector of the excitation current density derived from the multi-turn helical coil with the exciting current is modeled as:^67^

(22) $${{\bf{J}}_{\mathrm{e}}} = {I \over S}{{\bf{e}}_{{\mathrm{coil}}}} = {{N{I_{{\mathrm{coil}}}}} \over S}{{\bf{e}}_{{\mathrm{coil}}}}$$

where *I* is the total current (A) passing through the coil, *N* is the number of coil turns, *I*_coil_ is the current flowing through the single-coil loop, *S* is the cross-sectional area of the helical coil, and **e**_coil_ is the unit-vector tangent to the excitation coil.

#### Heat Transfer in Skin Tissue

To model the heat transfer in the skin tissue, we employed the following bioheat transfer equation:^68^,^69^

(23) $$\rho {C_{\mathrm{p}}}{{\partial T} \over {\partial t}} + \nabla \cdot \left({ - k\nabla T} \right) = {Q_{{\mathrm{nano}}}} + {Q_{{\mathrm{blood}}}} + {Q_{{\mathrm{met}}}}$$

where the first part is related to heat accumulation in the skin tissue during treatment time *t* (s), *C*_p_ (J/kg/K) is the specific heat of the tissue, *ρ* (kg/m^3^) is the tissue density, and *T* (K) is the current tissue temperature. The second part describes the conduction heating of the skin tissue with thermal conductivity *k* (W/m/K). The cooling effects of blood with perfusion *ω*_b_ (1/s) through the skin tissue are governed by:^11^

(24) $${Q_{{\mathrm{blood}}}} = {\rho _{\mathrm{b}}}{C_{\mathrm{b}}}{\omega _{\mathrm{b}}}\left({{T_{\mathrm{b}}} - T} \right)$$

Moreover, *Q*_met_ (W/m^3^) is a metabolic heat component produced by cell metabolism processes occurring in living tissues, and *Q*_nano_ is the heat loss dissipated in Fe_3_O_4_@Au MNPs, calculated using the theoretical formulations described in the upcoming section. The model assumes constant parameter values *ω*_b_ = 1.34∙10^–2^ 1/s, *Q*_met_ = 1.83 kW/m^3^ (see Table 2). Moreover, for simplicity, the model ignores the effects of resistive heating from copper coil windings, as described in.^25^,^70^

**Table 2:** Material Properties for All Modeled Objects

Materials	Blood^71^	Cancerous Skin Tissue (Muscle)^31^,^32^,^71^,^72^	Normal Skin Tissue (Skin)^71^	Copper Coil^70^	Air^71^
Domain Selection	Domains 1, 2	Domain 1	Domain 2	Domain 3	Domain 4
Density *ρ*, *ρ*_b_ (kg/m^3^)	1050	1090	1109	8700	1
Heat capacity *C, C*_b_ (J/kg/K)	3617	3421	3391	385	1004
Thermal conductivity *k* (W/m/K)	0.52	0.563	0.372	400	0.03
Heat transfer rate HTR (mL/min/kg)	10^4^	235	106	–	–
Blood perfusion rate *ω*_b_ (1/s)	–	1.34×10^–2^	1.96×10^–3^	–	–
Heat generation rate HGR (W/kg)	–	12	1.65	–	–
Metabolic heat generation *Q*_met_ (kW/m^3^)	–	13.08	1.83	–	–

The initial temperature for all domains was set to *T*_0_ = 37°C and the thermal insulation at the outer boundaries of the computational area was selected as follows:

(25) $${\bf{n}} \cdot \left({ - k\nabla T} \right) = 0$$

The convective heat flux at the skin surface was selected as follows:^73^

(26) $${\bf{n}} \cdot \left({ - {k_{{\mathrm{skin}}}}\nabla T} \right) = h\left({{T_{{\mathrm{ext}}}} - T} \right)$$

where *h* = 3.6 W/(m^2^∙K)^74^ is the heat transfer coefficient from the skin tissue surface to the outside, and *T*_ext_ = 37°C = 310.15 K is the external temperature at the outer edge of the treated tissue.

#### Mechanism of Heat Dissipation of Fe_3_O_4_@Au MNPs

The heat generated by the GMNPs due to the external alternating magnetic field (AMF) can be predicted using the linear response theory (LRT) proposed by Rosensweig:^61^,^62^

(27) $${Q_{{\mathrm{nano}}}} = {\mathrm{\pi }}{{\mathrm{\mu }}_0}{\chi _0}H_0^2f \left[{ {{2{\mathrm{\pi }}f\tau } \over {1 + {{\left({2{\mathrm{\pi }}f\tau } \right)}^2}}} } \right]$$

where *H*_0_ represents the amplitude of the external AMF (A/m) with frequency *f* (Hz), and *χ*_0_ is the static equilibrium magnetic susceptibility given by:^75^

(28) $${\chi _0} = {\chi _{\mathrm{i}}}{3 \over {\dot \xi }}\left[{{\mathrm{coth}} \left({\xi - {1 \over \xi }} \right)} \right]$$

where *ξ* and *χ*_i_ represent the dimensionless Langevin parameter and initial susceptibility, respectively, namely:

(29) $$\xi = {{{{\mathrm{\mu }}_0}{M_{\mathrm{d}}}{V_{\mathrm{m}}}{H_0}} \over {{{\mathrm{k}}_{\mathrm{B}}}T}}$$

(30) $${\chi _{\mathrm{i}}} = {{{{\mathrm{\mu }}_0}\phi M_d^2{V_{\mathrm{m}}}} \over {3{{\mathrm{k}}_{\mathrm{B}}}T}}$$

where k_B_ = 1.38×10^–23^ J/K is the Boltzmann constant, *M*_d_ (A/m) is the domain magnetization of the MNP core, and *ϕ* = *M*_s_/*M*_d_^76^ is the volume fraction of the magnetic element in the Fe_3_O_4_@Au MNP fluid, where *M*_s_ (A/m) is the saturation magnetization. The effective relaxation time is defined as:^62^,^77^

(31) $${1 \over {{\tau _{{\mathrm{eff}}}}}} = {1 \over {{\tau _{\mathrm{N}}}}} + {1 \over {{\tau _{\mathrm{B}}}}}$$

where *τ*_N_ and *τ*_B_ are the Neel and Brownian relaxation time constants:^78^,^79^

(32) $${\tau _{\mathrm{N}}} = {{\surd {\mathrm{\pi }}} \over 2}{\tau _0}{{{\mathrm{exp}}\left({\mathrm{\Gamma }} \right)} \over {\surd {\mathrm{\Gamma }}}}$$

(33) $${\tau _{\mathrm{B}}} = {{3\eta {V_{\mathrm{h}}}} \over {{{\mathrm{k}}_{\mathrm{B}}}T}}$$

where τ_0_ = 10^–9^ s is the attempt time, *η* is the nanofluid viscosity (Pa∙s), *K* is the magnetic crystalline anisotropy constant, and Γ is a parameter determined as follows:

(34) $$\Gamma = {{K{V_{\mathrm{m}}}} \over {{{\mathrm{k}}_{\mathrm{B}}}T}}$$

Moreover, for spherical particles, *V*_m_ and *V*_h_ represent the volumes of the magnetic core with diameter *D*_core_ and the whole Fe_3_O_4_@Au MNP with hydrodynamic coating of thickness *d*_shell_, respectively:

(35) $${V_{\mathrm{m}}} = {{\mathrm{\pi }} \over 6}{D^3}$$

(36) $${V_{\mathrm{h}}} = {{\mathrm{\pi }} \over 6} {\left({{D_{{\mathrm{core}}}} + 2{d_{{\mathrm{shell}}}}} \right)^3}$$

The heat dissipated by the Fe_3_O_4_@Au MNPs, defined by Equation (27), was computed for the Fe_3_O_4_@Au MNPs properties listed in Table 1, as *Q*_nano_ = 214 kW/m^3^. The value of this parameter is treated as a constant heat source and substituted into Pennes equation (23).

#### Estimation of Tumor Damage

The Arrhenius kinetic model was employed to predict the fraction of tumor damage.^80^,^81^ First, the Arrhenius integral or Arrhenius damage index is computed using the following formula:

(37) $$\Omega \left({x, y, z, t} \right) = {A_{\mathrm{f}}}\mathop \smallint \limits_0^t {\mathrm{exp}}\left[{{{ - {E_{\mathrm{a}}}} \over {{\mathrm{R}}T\left({x, y, z, t} \right)}}} \right]{\mathrm{d}}\tau $$

where *E*_a_ = 6.27×10^8^ J/mol is the activation energy, *A*_f_ = 3.1×10^98^ s^–1^ is the frequency factor,^82^ and R = 8.3144598 J/(mol∙K) is the universal gas constant. The necrotic fraction of the tumor is given by:^81^,^83^

(38) $$\theta \left({x, y, z, t} \right) = 1 - \exp \left[{\Omega \left({x, y, z, t} \right)} \right]$$

## Simulations Results

This section presents the simulation results of the model formulations developed for the skin cancer problem statement. These results will be analyzed and interpreted in correlation with the findings described in the literature. We will discuss the practical visualization of the proposed treatment protocol for skin therapy with Fe_3_O_4_@Au MNPs. The complete implementation of coupled electro-thermal formulations in COMSOL Multiphysics software to create a geometry to predict the fraction of tumor damage is demonstrated.

### Adding Physics, Studies, and Geometry Construction of the Model

To solve the problem of the current study using COMSOL Multiphysics software, the physics of the analyzed skin cancer model, described by Equations (1)–(38), was first assimilated. Next, three studies, namely, the stationary, time-dependent, and frequency domains, were added to simulate the steady-state, time-dependent, and frequency domains of the model.

**Figure 3:**
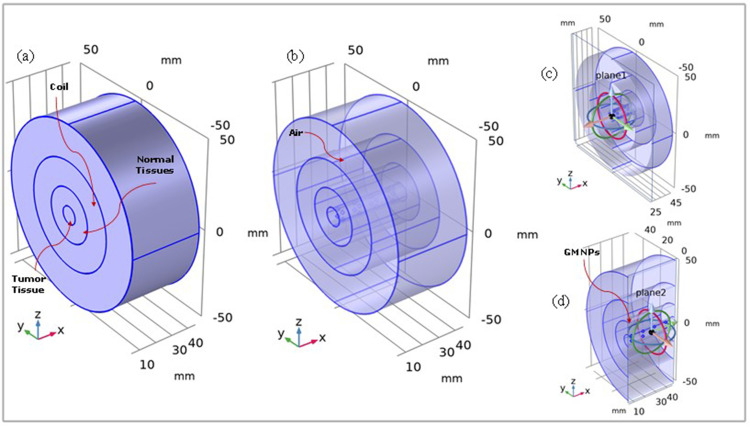
Geometry of the analyzed model: (**a**) cylindrical model; (**b**) transparent view; (**c**) semi cross sectional view (plane 1); (**d**) half region of the geometry (plane 2) with Fe_3_O_4_@Au MNPs in the tumor tissue layer.

The model geometry was constructed by adding physics and studies. The considered model included four coaxial cylinders of different heights and radii, representing the tumor with Fe_3_O_4_@Au MNPs, normal skin tissue, copper coils, and external air. All cylinders had bases centered at the origin point (0,0,0) as shown in Figure 3. The details of the modeled objects and their dimensions are presented in Table 3.

**Table 3:** Details of Objects Geometry and Their Dimensions

Objects/Domains	Radius (mm)	Height (mm)
Domain 1 (tumor with Fe_3_O_4_@Au MNPs)	5	30
Domain 2 (normal skin tissue)	15	30
Domain 3 (cooper coil)	30	40
Domain 4 (external air)	50	40

### Adding Materials to the Geometry Objects

Subsequently, we filled the domains with these three materials. We added muscle material properties to simulate cancerous skin tissue (Domain 1) and skin material properties to simulate normal skin tissue (Domain 2). Copper material was added to Domain 3 and air was added to Domain 4. In this study, we assumed that both normal and cancerous tissues had almost identical physical properties. Details of the material properties are presented in Table 2. The tissue parameter values were obtained from the IT’IS tissue database.^71^ The blood properties (equipped with index b) and copper coil parameters^70^ are listed in Table 2.

### Mesh Construction of the Model

**Figure 4:**
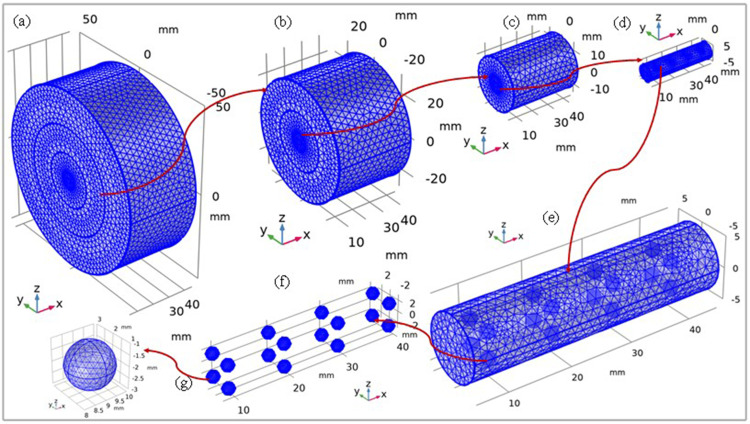
Meshing of the model: (**a**) transparent view of whole model; (**b**) the coil with normal and tumor tissues; (**c**) the normal and tumor tissues; (**d**) the tumor with Fe_3_O_4_@Au MNPs; (**e**) zoomed view of (**f**) gold iron-oxide MNPs; (**g**) one zoomed Fe_3_O_4_@Au MNP.

Subsequently, the mesh of the model was constructed using a finer mesh mode. Tetrahedral elements were selected as the basic mesh element type. A completely used mesh is shown in Figure 4. The details of the number of elements in the mesh are listed in Table 4.

**Table 4:** Types and Number of Elements in the Mesh

Types of Elements	Tetrahedral Elements	Vertex Elements	Edge Elements	Boundary Elements	Minimum Element Quality
**Number of elements**	10836	176	548	1992	0.2304

### Multi-Turn Coil Design and Simulation of Magnetic Field

**Figure 5:**
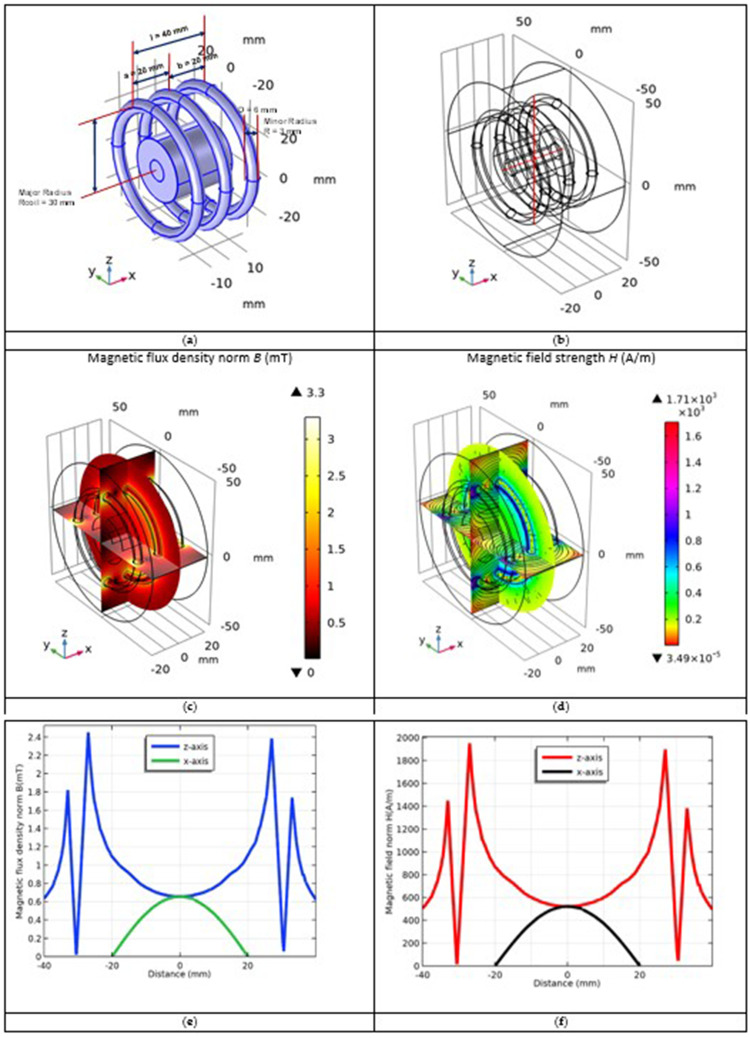
Multi-turn coil simulation results: (**a**) three-turn coil design surrounding the tumor and normal tissues; (**b**) selected line paths along x-, and z-direction crossed the excitation coil; (**c**) magnetic field B (mT); and (**d**) magnetic field strength H (A/m) distributions inside the excitation coil with current I_coil_ = 15 A and frequency f = 571 kHz; (**e**) B-norm; and (**f**) H-norm curves along x- and z-directions.

The source of the magnetic field, that is the current-carrying coil, was designed as shown in Figure 5a. We constructed a 3-turn copper coil with major radius *R*_coil_ = 30 mm and minor radius *R* = 3 mm of coil winding. The frequency of the coil was assumed to be *f* = 571 kHz and the current was set to *I*_coil_ = 15 A. The properties of the excitation coils are presented in Table 3.

First, the applied external AC magnetic field of the excitation coil with current *I*_coil_ = 15 A and frequency *f* = 571 kHz was simulated. To analyze the norm value of the magnetic field strength versus distance, two line paths along the *x*- and *z*-direction were considered, as shown in Figure 5b.

The magnetic field induction had a maximum value of *B*_max_ = 4.94 mT near the coil windings, as shown in Figure 5c. At the coil center, where the tumor tissue layer with Fe_3_O_4_@Au MNPs are placed, the magnetic field has a quasi-uniform distribution with non-zero value of about *B*_0_ = 0.7 mT.

Magnetic field strength norm at frequency 571 kHz has been simulated in Figure 5d. In the coil center the magnetic field strength norm has a value of *H*_0_ = 750 A/m. The *B*-curve and *H*-curve along *x*- and *z*-axes are shown in Figure 5e and f, respectively.

**Figure 6:**
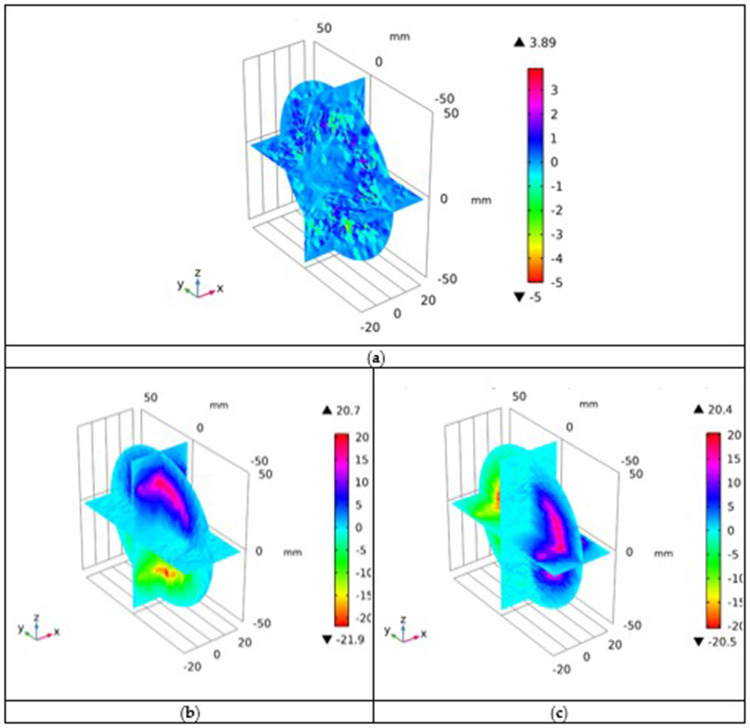
Distributions of magnetic vector potential in the case of: (**a**) *A_x_*; (**b**) *A_y_*; and (**c**) *A_z_* components (*A*-values are given in μWb/m).

Subsequently, the components of the electromagnetic vector potential **A** = (*A_x_, A_y_, A_z_*) Wb/m were simulated as shown in Figure 6a–c, respectively. The distribution of *A_x_* component varied throughout the domain between positive and negative values, with a mixed distribution. The behaviors of *A_y_* and *A_z_* components differed from those of the component *A_x_*. In the case of *A_y_* component, the values of the potential are positive in the upper half-plane and negative in the lower half-plane of the *z*-axis. For *A_z_* component, the values of the potential are positive in the right-half plane and negative in the left-half plane of the *y*-axis. This result indicates that the greater the potential distribution in the tumor region, the greater the tumor damage owing to the uniform heating.

Along with computational analysis, it is now time to determine the analytical treatment of the magnetic field distribution by the coil. The analytical solution for the on-axis magnetic field was adopted from:^29^,^72^

(40) $${B_z} = {{{B_{z\,{\mathrm{max}}}}} \over 2}\left[{{{{l \over 2} - z} \over {\sqrt {{{\left({z - {l \over 2}} \right)}^2} + {R^2}} }} + {{{l \over 2} + z} \over {\sqrt {{{\left({z + {l \over 2}} \right)}^2} + {R^2}} }}} \right] = {{{{\mathrm{\mu }}_0}N{I_{{\mathrm{coil}}}}} \over {2l}}\left[{{{a - z} \over {\sqrt {{{\left({z - a} \right)}^2} + {R^2}} }} + {{b + z} \over {\sqrt {{{\left({z + b} \right)}^2} + {R^2}} }}} \right]$$

where *B_z_*_max_ = μ_0_*nI*_coil_ = μ_0_*NI*_coil_/*l, l* = 40 mm is the length of the coil, a and b are half the length of the coil, as shown in Figure 5a.

**Figure 7:**
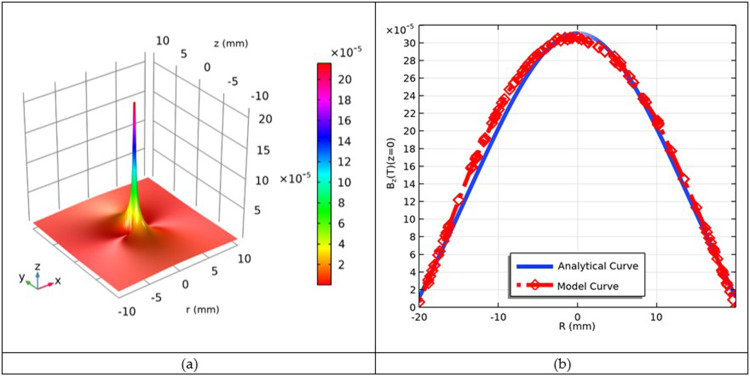
Analytical solution for on-axis magnetic flux density *B_z_*(*z,r*) given in teslas (T) obtained from Equation (39) (**a**); and (**b**) comparison of computational and analytical *B*-curves.

The analytical solution was plotted using the COMSOL Multiphysics software, as shown in Figure 7a. The peak magnetic field induction reaches a maximum value of 0.32 mT that decreases as we move from the peak to the ground, where a zero value is observed. The solution converges at the center.

To investigate the accuracy of our computed results for the multi-turn coil, we compared them with the analytical solution for the 3-turn coil, as shown in Figure 7b. The computational and analytical curves were in good agreement, thus confirming the accuracy of the computed results.

### Simulation of the Temperature Distribution

**Figure 8:**
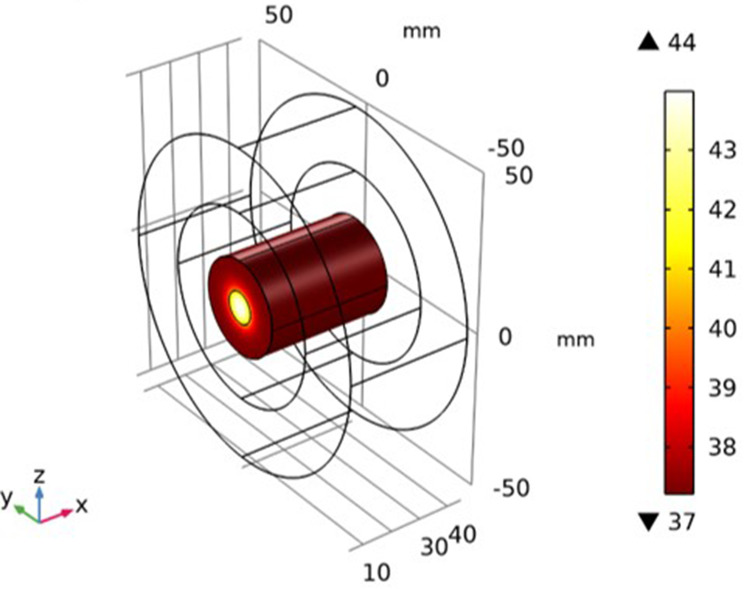
Prediction of temperature distribution inside skin tumor tissue after 60-minutes of treatment.

It is assumed that the Fe_3_O_4_@Au MNPs were injected into the tumor, and we waited for nearly 24 hours to distribute the Fe_3_O_4_@Au MNPs throughout the tumor. Now the external magnetic field has been switched on. The Fe_3_O_4_@Au MNPs began to vibrate under the influence of an external magnetic field. The heat is dissipated by vibrating particles under the influence of dipole moments and Neel-Brownian relaxation effects. This heat increases the temperature of the tumor. The resulting temperature distribution inside the tumor is shown in Figure 8. The temperature was elevated to 42.3°C from the normal temperature of 37°C of the tumor tissue. The temperature is maximum at the central region of the tumor and decreases when moving away from the center towards the outer boundary of the tumor tissue.

**Figure 9:**
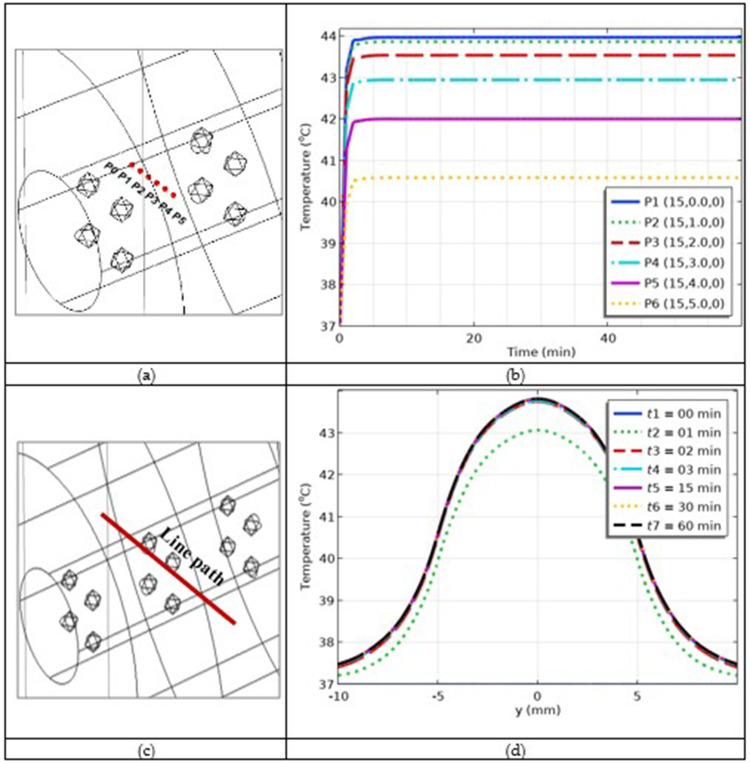
(**a**) Visual display of selected points; (**b**) temperature versus time profiles at the selected points; (**c**) visual display of selected arc length; and (**d**) temperature versus time profiles along the selected line path.

For quantitative analysis, we selected six points, P1, P2, P3, P4, P5 and P6 in the skin tissue, whose visual clues are shown in Figure 9a. The temperature versus time curves at these selected points are shown in Figure 9b. The temperature at point P1 was the maximum and that at point P6 was the minimum. Therefore, the temperature decreased from the maximum value at the center to the minimum value at the outer boundary of the tumor. The temperature increased to its maximum value within the first five minutes of heating, and this value was maintained until the end of heating. Next, to investigate the variation in the temperature versus space coordinates; a line path was selected, as shown in Figure 9c. The temperature versus arc length curves for different times are shown in Figure 9d. As the time increased, the temperature curves increased. The temperature was maximal at the tumor center and minimal at the outer boundary of the tumor.

### Simulation of the Fraction of Cancer Damage

**Figure 10:**
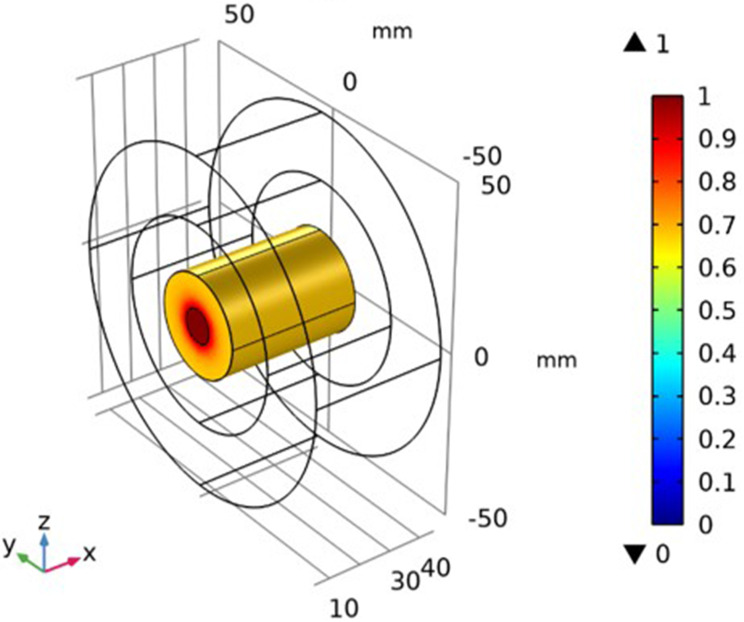
Prediction of tumor damage fraction inside skin tumor tissue after 60 minutes of treatment.

**Figure 11:**
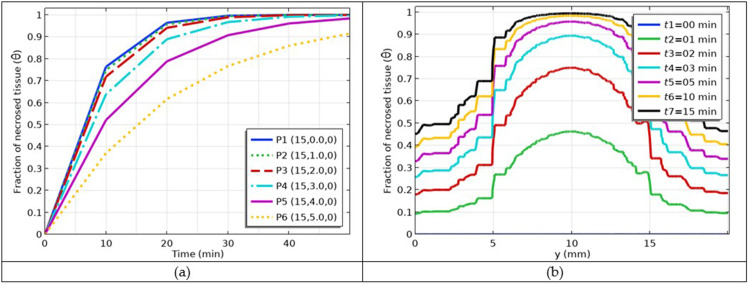
(**a**) Estimation of tumor damage fraction versus time at the selected points; and (**b**) temperature profiles along selected line path with the increasing treatment time.

Currently, we are in a position to predict tumor damage. The fraction of tumor damage (*θ*) was predicted to be 90–99%, as shown in Figure 10. The tumor damage was maximal at the tumor center because the heating was maximal at this location. The damage was minimal at the outer boundary of the tumor. We succeeded in achieving maximum tumor damage. For quantitative analysis, we selected the same points as those used previously in the temperature estimation case. The fraction of tumor damage versus time curves at the given selected points is shown in Figure 11a. The visual clues of the points are shown in Figure 9a. The simulations show that with increasing heating time, the fraction of tumor damage increases and is maximum at the center and minimum at the outer boundary of the tumor. The fraction of tumor damage versus selected path is plotted in Figure 11b, where a visual clue of the line path is shown in Figure 9c. The fraction of tumor damage curves shifted to a higher damage fraction with increasing time. The damage is maximum at the center and minimum at the outer boundary of the tumor tissue. The non-smoothness of the curves was due to damaged tissue.

### Simulation of the Impact of Varying Blood Perfusion

**Figure 12:**
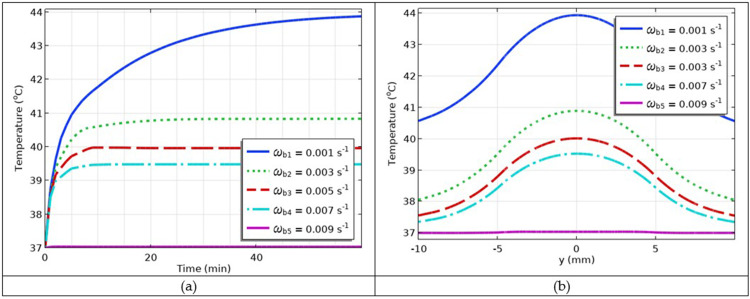
(**a**) The temperature versus time profiles at different values of blood perfusion; (**b**) temperature versus *y*-coordinate profiles at different values of blood perfusion.

To investigate the impact of different blood perfusion rates on hyperthermia, temperature versus time and temperature versus space coordinates at perfusion rates *ω*_b1_ = 0.001 1/s, *ω*_b2_ = 0.002 1/s, *ω*_b3_ = 0.003 1/s, *ω*_b4_ = 0.004 1/s, *ω*_b5_ = 0.005 1/s were simulated as shown in Figure 12a and b, respectively. The results showed that, as the blood perfusion rate increased, the temperature decreased. Physiologically, during hyperthermia, when body temperature increases, blood perfusion decreases and ultimately moderates body temperature. Otherwise, patients may experience severe discomfort and injuries.

### Simulation of the Impact of Varying Metabolic Heat Generation Rates

Metabolic heat generation is a process in which natural heat from human tissues is produced during physical work. During hyperthermia, metabolic heat is generated from the body tissues and affects the overall body temperature. Here, we plug several values of metabolic heat into the model and investigate its impact on the temperature distribution. The selected values are *Q*_met1_ = 1 kW/m^3^, *Q*_met2_ = 2 kW/m_3_, *Q*_met3_ = 3 kW/m^3^, *Q*_met4_ = 4 kW/m^3^, *Q*_met5_ = 5 kW/m^3^.

**Figure 13:**
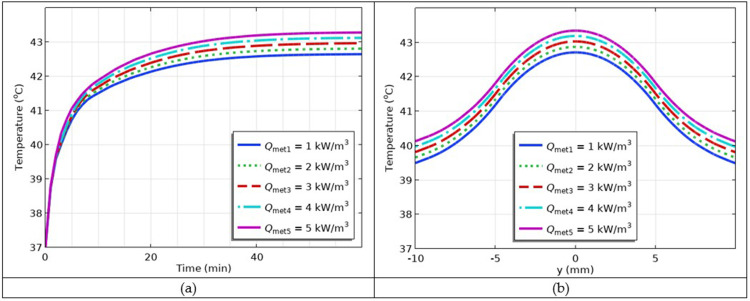
(**a**) The temperature versus time profiles at different values of metabolic heat generation; (**b**) temperature versus *y*-coordinate profiles at different values of metabolic heat generation.

The simulation results are shown in Figure 13a and b. The results showed that as the metabolic heat generation increased, the body temperature also increased, which was directly proportional to the temperature. This contributes to the overall body temperature.

### Simulation of the Impact of Heat Generation Produced by MNPs

**Figure 14:**
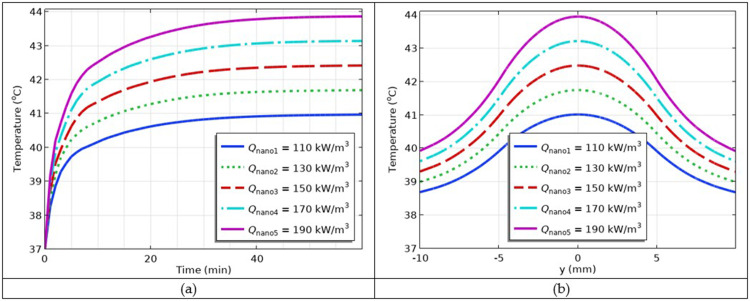
(**a**) The temperature versus time profiles at different values of heat generation due to Fe_3_O_4_@Au MNPs; (**b**) temperature versus *y*-coordinate profiles at different values of heat generation cased by Fe_3_O_4_@Au MNPs.

Evidently, more heat results in a higher temperature. To justify this statement through the model, the different values of heat generated by the MNPs were selected *Q*_nano1_ = 110 kW/m^3^, *Q*_nano2_ = 130 kW/m^3^, *Q*_nano3_ = 150 kW/m^3^, *Q*_nano4_ = 170 kW/m^3^, *Q*_nano5_ = 190 kW/m^3^ and the resulting simulations by plugging these values into the model are shown in Figure 14a and b, respectively. The results showed that if the MNPs dissipated more heat, higher temperatures would be obtained. Higher temperatures are generally used in ablation therapy.

### Simulation of the Impact of Varying Blood Densities

**Figure 15:**
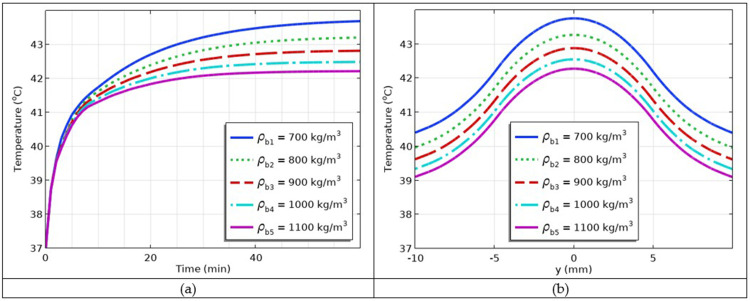
(**a**) Temperature versus time profiles at different values of blood densities (**b**) Temperature versus *y*-coordinate profiles at different values of blood densities.

The blood density plays a major role in hyperthermia. More and less dense blood differentially affect thermal therapies. Five different blood densities, *ρ*_b1_ = 700 kg/m^3^, *ρ*_b2_ = 800 kg/m^3^, *ρ*_b3_ = 900 kg/m^3^, *ρ*_b4_ = 1000 kg/m^3^, *ρ*_b5_ = 1100 kg/m^3^, were selected and plugged into the model. The corresponding results are given in Figure 15a and b. The results showed that as blood density increased, temperature decreased. Thus, the blood density inversely affects the hyperthermia temperature.

### Simulation of the Impact of Varying Blood Heat Capacities

**Figure 16:**
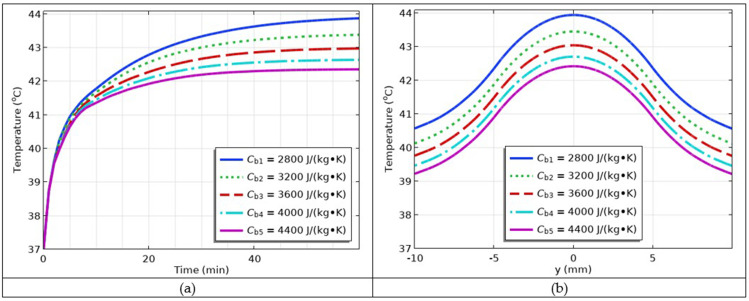
(**a**) The temperature versus time profiles at different values of blood heat capacities; (**b**) temperature versus *y*-coordinate profiles at different values of blood heat capacities.

The impact of the last parameter investigated in the model was the blood heat capacity. The values of the blood heat capacity were as follows: *C*_b1_ = 2800 J/(kg∙K), *C*_b2_ = 3200 J/(kg∙K), *C*_b3_ = 3600 J/(kg∙K), *C*_b4_ = 4000 J/(kg∙K), *C*_b5_ = 4400 J/(kg∙K). The simulations are shown in Figure 16a and b, respectively. The results showed that, as the blood heat capacity increased, the temperature decreased. Therefore, this parameter also inversely affects hyperthermia temperature.

### Simulation of the Additional Parameters

**Figure 17:**
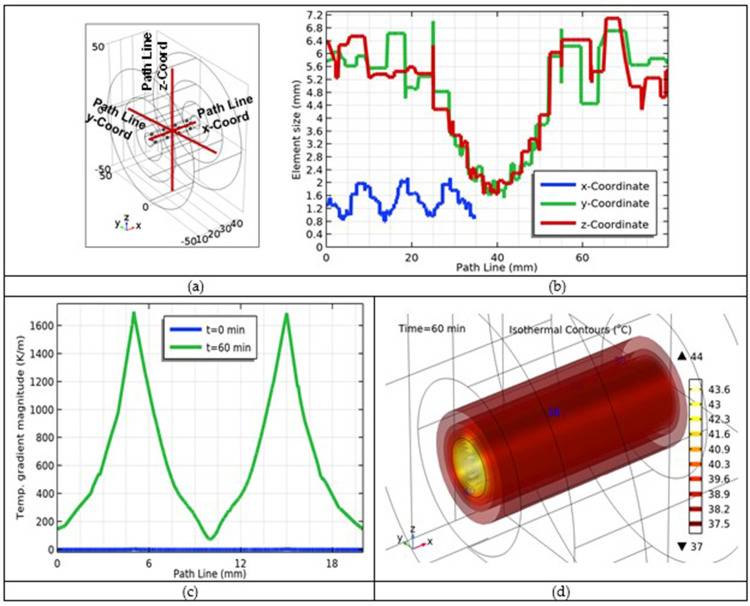
(**a**) Selected line paths along the Cartesian coordinates; (**b**) Element sizes versus selected line paths; (**b**) temperature gradient versus line path; and (**c**) ISO-surfaces after 60-minute treatment.

Next, we plotted additional graphs for some other important parameters. Another line paths along the individual Cartesian coordinates were selected as shown in Figure 17a. A plot of the element sizes versus distance along these line paths is shown in Figure 17b. The results showed that the element size was minimal when the center element size increased when moving away from the center, and was maximum value at the outer edges of the tumor. In next step, the temperature gradient versus the line path simulated from the initial time *t* = 0 min to the final point of treatment at *t* = 60 min, as shown in Figure 17c. This shows that the fluctuations in the graph increased at the end of given path. However, its behavior was linear during the initial phase. Moreover, an isothermal contours (ISO-surfaces) after 60-minute period of treatment are shown in Figure 17d. The temperature at the cancerous tissue is higher and minimum in the normal tissue, and the heat flux is almost uniform along the entire skin tissue.

## Discussions

In the current study, we attempted to provide an alternative skin cancer treatment protocol in contrast to in vivo and in vitro studies. The experiments are subject to time and cost constraints. We performed a cold test based on computational analysis of the coupled modeling framework of the thermal therapy procedures involved in the given electro-thermal problem. The modeling and simulation procedure starts with a situation, in which Fe_3_O_4_@Au MNPs are injected into the cancer-affected tissue of the body. The Fe_3_O_4_@Au MNPs can be injected into the tumor using different-gauge needles, which are generally used in clinical settings. The Fe_3_O_4_@Au MNPs are generally distributed uniformly inside the tumor after approximately 24 hours. Subsequently, the external magnetic field is switched on. The Fe_3_O_4_@Au MNP begin to vibrate, and during their back and forth motion in the form of a dipole moment, the heat is dissipated. The heat generated by the Fe_3_O_4_@Au MNPs under the influence of the Neel and Brownian relaxation effects kills cancerous cells. The process of tissue damage is still not completely understood; however, most researchers believe that tumor cells have been killed by cytotoxic and coagulation effects.

There are four main processes involved in thermal therapy after the diffusion of MNPs: 1) the external magnetic field is working and 2) heat is generated by the Fe_3_O_4_@Au MNPs. 3) The generated heat is transferred to the tissue, and 4) the tumor is damaged due to generated heat. To develop the modeling framework for the above processes, first, the magnetic field of the current-carrying coil was modeled using Ampere’s law associated with the 3-turn coil current. Second, the Rosensweig’s model is employed to model the heat-generation mechanism. Third, to model heat transfer in the tissue, the Pennes bioheat transfer model is considered. It has the potential to capture the main features of heat transfer in the tissue along with heat transfer in blood vessels associated with blood perfusion. Fourth, the Arrhenius kinetic model is used to predict the tumor damage. These models, in the form of partial differential equations, are coupled using the Multiphysics coupling model tool available in the COMSOL software. The selection of models is difficult. However, we selected the one that best portrayed the physical phenomena involved in the therapy.

The coupling models can be solved using different numerical methods, such as finite difference, finite volume, finite element, boundary element, Galerkin, and collocation methods. The selection of a method to solve the modeling framework of our problem is very difficult. However, based on certain characteristics, the finite element method (FEM) was selected. This method solves PDEs into a weak form and then converts them into a system of linear equations. These equations were then solved for each node of the discretized mesh of the model. A FEM engine solves the equations not only at the node points, but also in the regions between consecutive nodes. The major advantage of the FEM in solving this problem is geometry flexibility. It is more powerful in handling complex geometric problems and solving them, not only at the surface but also at the entire domain of the model. As this method already exists in COMSOL Multiphysics software, we took advantage of it and solved the given electro-thermal problem. The mesh was based on tetrahedral elements.

The infused nanofluid inside the tumor is collected right after the needle tip and form the cavity. This cavity formation is not still understood completely. It might gain any irregular shape. The pressure from injecting needle has been shifted to the nanofluid gathered at the cavity. This pressure causes the MNPs to disperse in the entire tumor insterstitium. That is why the simulation results for concentration of diffusion is maximum at the center to minimum at the outer boundary of tumor. Some of the researchers also believe that this diffusion is caused by the back and forth motion of the tissue in the form of travelling waves. The MNPs concentration is symmetrical along the vertical line. Such a curve is very close to Gaussian distribution curve. Now, if the switch of the external magnetic field is on, the lines of magnetic force cross the tumor region and stir the MNPs. The friction between spinning MNPs and tissue layers cause to produce heat and thus elevate the temperature of tissue. Now, the heat will be maximum in those regions of tumor where concentration of MNPs will be maximum at the center of the tumor. That is why the predicted temperature from simulations is maximum at the center and minimum at the outer boundary of the tumor, where minimum MNPs concentrating was observed. The higher is the temperature at any location of the tumor, the higher will be tumor damage. That is why; the fraction of tumor damage in our simulated results is maximum at the center and minimum at the outer boundary of the tumor. Ideally, the interface at the tumor-normal tissue is at 37°C. There is probability of penetration of small fraction of MNPs from cancerous tissue to the normal tissue. From macroscopic scale this penetration fraction is negligible.

The effectiveness of heat in damaging tumor tissue depends on numerous elements, namely: a) *Temperature and duration of exposure*: Higher temperatures (over 45°C) lead to rapid tumor destruction via necrosis, even as lower temperatures (40–42°C) are commonly used in hyperthermia to sensitize tumors to other treatments. The period of warmth exposure is equally critical; longer periods at mild temperatures (40–45°C) growth the chance of cellular harm. b) *Tumor type and blood flow*: Tumors with complex vascularity and bizarre blood flow are more vulnerable to warmness-brought about harm because the dearth of efficient blood flow prevents warmth from dissipating and consequences inside the build-up of thermal power within the tumor tissue. Hypoxic tumors (with low oxygen levels) regularly reply better to warmness treatment due to the fact their impaired blood flow makes them less capable of deplete warmth. c) *Heat distribution*: Tumors are regularly heterogeneous in nature, which means that warmth distribution is not uniform across the tumor. Uneven heating can bring about quantities of the tumor being under heated or inadequately dealt with. Technologies, which includes MRI-guided thermal remedy or thermography, are used to display temperature distribution and optimize treatment delivery. d) *Clinical considerations side effects and risk to healthy tissue*: One of the challenges of the usage of warmth to treat tumors is the potential hazard to surrounding healthy tissues. Although thermal safety strategies are frequently hired, there may be nonetheless the hazard of collateral harm to close by wholesome tissue, particularly in sensitive organs. e) *Combination with other therapies*: Heat is often mixed with chemotherapy, radiation therapy, or surgical procedure. In unique, hyperthermia is a powerful adjuvant to radiation therapy, making tumor cells more susceptible radiation-prompted DNA harm. Heat is a powerful device for tumor destruction, leveraging mechanisms which include protein denaturation, DNA damage, and cell membrane disruption. The one of a kind sorts of heat remedy, which include hyperthermia, radiofrequency ablation, and microwave ablation, every have their place inside the remedy of tumors, particularly whilst combined with other remedies. Factors like temperature, exposure time, and tumor vascularity drastically have an effect on the fulfillment of heat-based treatments. Although warmness treatments may be noticeably powerful, cautious making plans and monitoring are required to make certain highest quality tumor destruction while minimizing harm to surrounding healthful tissues.

The vibration of Fe_3_O_4_@Au MNPs depends on the applied magnetics flux density. During the vibration of the Fe_3_O_4_@Au MNPs striking the tumor tissue, the heat is dissipated. While tuning the externally applied magnetic fields through the amplitude and frequency, it should be noted that most of the magnetic line forces cut the cancerous part of the tissue. Heat dissipation due to MNPs alleviated the tissue temperature. This may be a 5°C increase in temperature from the normal body temperature. During the therapeutic procedure, one should be careful that the tumor is not roasted during overheating. Elevated temperatures kill cancer cells. Normal tissues surround the tumor tissue, and their walls are adjacent. Therefore, there is a chance of damage to some parts of normal tissue. Temperature elevation during thermal therapy affects many other biological parameters associated with tissue. Elevated temperatures are naturally moderated by perfusion in the blood vessels associated with the tissue. The density of blood and heat capacities also changed under the influence of temperature elevation. Blood perfusion, blood density, and blood heat capacity vary inversely with temperature, whereas metabolic heat and heat generated by MNPs vary directly at elevated temperatures. Elevated temperatures reduce the density of blood inside the vessels, which negatively impacts the human body and causes discomfort to the patient.

Although all the parameters involved in the bioheat Pennes equation are sensitive to the heat generated by MNPs, yet the blood perfusion rate is the most sensitive parameter. The small variation in the values of blood perfusion may results into very large and abrupt changes in the output heat and ultimately may shoot the elevated temperature in the tissue.

Cancer is a complex disease involving an irregular geometry. Such geometries can be handled effectively if finite element method (FEM) is used instead of analytical approaches. The FEM presents the geometric flexibility of the problems. In the current study, we ignored the microscopic interaction of the skin tumor with the surroundings and only considered a cylindrical tumor of fourth grade, which is fully developed in the skin. This work is novel in the following ways: 1) Contrary to the usage of traditionally used MNPs, such as iron oxide MNPs, we have employed another latest synthesized type of biocompatible gold-coated magnetite nanoparticles (Fe_3_O_4_@Au MNPs). In vivo and in vitro experimental studies have demonstrated their efficacy in cancer treatment. Moreover, the real sizes of the magnetic core and the gold shell of used nanoparticles assumed in numerical simulation were determined based on measurements performed by direct current plasma–atomic emission spectroscopy (DCP-AES) given in the literature.^63^ To the best of the authors’ knowledge, there are no similar in silico studies using such a precise method for estimating the sizes of gold-coated magnetite nanoparticles (Fe_3_O_4_@Au MNPs). 2) We created a unique geometry of the model using COMSOL Multiphysics software, where objects are taken in cylindrical form and represent the tumor tissue, normal tissue, copper coil, and external air. The heating source Fe_3_O_4_@Au MNPs have been generated as spheres. Therefore, the geometry created in our study is an approximate prototype of an actual biological phenomenon. 3) The FEM was used to create a mesh for the model. This method possesses many advantages in dealing with such geometries over the other known methods like the finite difference method (FDM), finite volume method (FVM), and boundary element method (BEM). 4) Before solving the whole physics, we coupled all the physics of the model, what is not an easy task in COMSOL Multiphysics. 5) The impact of various important parameters contributing to hyperthermia has been investigated using the proposed modeling and simulation framework. 6) Through this computational study, we succeeded in damaging 90–99% of the tumor, which is very close to the complete eradication of the tumor. 7) The *H*-curves and *B*-curves have been plotted from the magnetic field distribution generated by the 3-turn coil. 8) A comparison of analytical magnetic field curves with computational model curves is performed to show the accuracy of our computed results, and some additional parameters like thermal tumor tissue damage have also been simulated. Lastly, contrary to time-consuming and costly in vivo and in vitro studies, we have developed a computer-based pretreatment protocol, very helpful in planning magnetic hyperthermia treatment using Fe_3_O_4_@Au MNPs.

One of the limitations of the current study is, that we did not get the complete tumor damage. This is a limitation of computational studies that cannot ideally be identical to actual biological phenomena. Moreover, in real-time in vivo and in vitro studies, 100% tumor damage was not observed. Therefore, thermal therapy is integrated with other traditional therapies to obtain the desired results. Consequently, this limitation can be compensated for, if combined with radiotherapy, chemotherapy, immunotherapy, or surgery. The future will involve integrated therapies.

To enhance the therapeutic qualities of Fe_3_O_4_@Au MNPs in melanoma and other malignancies, more research is required that focuses on evaluating their in vivo and in vitro targeting efficiency. In addition, long-term cytotoxicity research must be conducted at greater depths, which helps to lessen these Fe_3_O_4_@Au MNP’s negative impact on healthy cells. All research findings offer a key recommendation for the creation of a thermal treatment improved by Fe_3_O_4_@Au MNPs. This study can offer a theoretical guide for current clinical therapy through a simulated study of the tissue temperature field. The results from the simulations have not yet been experimentally confirmed owing to condition constraints, necessitating further investigation.

## Conclusions

In this research, we examined the hyperthermia technique that mostly damage the tumor tissues and less damage the normal tissues of skin that is why it proves to be a more efficient technique. Temperature is maximum inside the tumor and minimum outside the edges. It ranges from 37°C to 42–43°C within 60 minutes of magnetic hyperthermia treatment. We have predicted the tumor damage is about 90–99%. However, if the tumor can regrow then it is better to couple this therapy with other therapies like laser therapy to kill the tumor permanently. The Fe_3_O_4_@Au MNPs proved to be more efficient for skin cancer therapy. By interjecting Fe_3_O_4_@Au MNPs, the tumor is more heated and thus destroyed. Electromagnetic heating and magnetic flux density are maximum near the coil because of the magnetic field and minimum at other regions. The temperature at the center of the tumor is maximum and it decreases in moving away from the center along the space coordinates. The tumor damage at the center of the tumor is maximum and it decreases in moving away from the center along the space coordinates. The magnetic flux density is uniformly crossing the skin tissue however, its concentration is maximum across the tumor that is we wanted to maximum heat the Fe_3_O_4_@Au MNPs. A well-behaved *H*-curve and *B*-curve from heating coil was obtained. The blood perfusion is more sensitive and varies inversely with changes in temperature. The metabolic heat generation varies directly with temperature. Tumor tissue temperature varies directly with heat generated by Fe_3_O_4_@Au MNPs. The blood density varies inversely with temperature. The blood heat capacities varies inversely with the temperature. It should be pointed that computational results have been compared with analytical results for coil in good agreement.
